# Rationale, Methods, Feasibility, and Preliminary Outcomes of a Transdiagnostic Prevention Program for At-Risk College Students

**DOI:** 10.3389/fpsyt.2019.01030

**Published:** 2020-02-25

**Authors:** Anne S. Burke, Benjamin G. Shapero, Andrea Pelletier-Baldelli, Wisteria Y. Deng, Maren B. Nyer, Logan Leathem, Leah Namey, Carrie Landa, Corinne Cather, Daphne J. Holt

**Affiliations:** ^1^ Department of Psychiatry, Massachusetts General Hospital, Boston, MA, United States; ^2^ Harvard Medical School, Boston, MA, United States; ^3^ Department of Psychiatry, University of North Carolina, Chapel Hill, NC, United States; ^4^ Department of Psychology, University of California, Los Angeles, Los Angeles, CA, United States; ^5^ Behavioral Medicine, Boston University, Boston, MA, United States

**Keywords:** resilience, prevention, self-compassion, college students, transdiagnostic, psychosis, depression, transition-aged youth

## Abstract

**Purpose:**

Early adulthood represents one period of increased risk for the emergence of a serious mental illness. The college campus provides a unique opportunity to assess and monitor individuals in this at-risk age group. However, there are no validated early detection programs that are widely implemented on college campuses. In an effort to address this gap, we designed and tested an early detection and prevention program tailored to college students. A transdiagnostic approach was employed because of evidence for shared risk factors across major mental illnesses.

**Design:**

Single arm, prospective study evaluating outcomes following a 4-week intervention.

**Method:**

Three in-person mental health screenings were conducted on the campus of one university. Undergraduate students with at least mildly elevated, self-reported levels of depressive or subclinical psychotic symptoms, who were not receiving treatment for these symptoms, were invited to participate in a 4-session workshop focused on increasing self- and other- awareness and emotion regulation using established mindfulness, self-compassion, and mentalization principles and experiential exercises. Symptoms, resilience-promoting capacities, and aspects of social functioning were assessed pre- and post- intervention.

**Results:**

416 students were screened and a total of 63 students participated in the workshop. 91% attended at least 3 of the 4 sessions. The majority of participants found the workshop interesting and useful and would recommend it to a friend. Significant pre-to-post reductions in symptoms (depression, anxiety, and subclinical psychotic symptoms, *p*s < 0.004) and improvements in resilience-promoting capacities (self-compassion and self-efficacy, *p*s < 0.006) and indices of social functioning (social motivation, activity, and a measure of comfort with the physical presence of others, *p*s < 0.04) were observed. Moreover, the significant increases in resilience-promoting capacities correlated with the reductions in affective symptoms (*p*s < 0.03).

**Conclusions:**

These findings suggest that an on-campus mental health screening and early intervention program is feasible, acceptable, and may be associated with improvements in resilience-related capacities and symptom reductions in young adults with non-impairing, subclinical symptoms of psychopathology. Follow-up work will determine whether this program can improve both shorter and longer-term mental health and functional outcomes in this at-risk population.

## Introduction

Many neuropsychiatric illnesses initially manifest during late adolescence and early adulthood (1, 2). Thus, it has been proposed that some resources allotted for the early detection and prevention of serious mental illness should focus on this developmental stage (3, 4). Support for this idea comes from recent studies reporting positive effects of psychosocial interventions [e.g., cognitive behavioral therapy (CBT)] delivered to at-risk individuals in this age group that appear to either prevent or delay the onset of syndromal illness or reduce the severity of emerging symptoms (5–11). However, to date, few early detection and intervention programs have been implemented in “real world” settings where a substantial number of people could benefit. This may be in part because many of the methods that have been used for research on early detection and prevention of psychopathology are too costly and/or lengthy to be widely implemented (12).

The college campus is a setting with a clear need for early intervention services, since the majority of college students experiencing symptoms of mental illnesses do not receive adequate clinical attention (13). This gap in care may be attributable to a variety of factors, including the limited resources available for mental health care at these institutions and stigma-related barriers to seeking help.

Studies of early detection and prevention in college students have primarily enrolled those with mild, subsyndromal symptoms of depression, anxiety, or anger (12). To date, no studies have focused on students with symptoms that are more closely linked to incipient serious mental illness (e.g., subthreshold psychotic symptoms, cognitive deficits, functional impairment). Given that the peak period of risk for the onset of psychotic disorders such as schizophrenia overlaps with the college years, it is surprising that early intervention efforts on college campuses have not focused on students who are at increased risk for these debilitating illnesses, particularly since psychotic disorders are associated with high levels of academic and overall disability (14–17) and elevated school drop-out rates (18).

Prodromal states of psychosis are most commonly identified using interview-based research instruments that require extensive training and time to administer; thus, they cannot be implemented broadly for mental illness screening in college students. However, symptoms associated with increased risk for serious mental illness can also be identified reliably using self-report questionnaires (19–21). The symptoms of schizotypy, “psychosis-like,” or “psychotic experiences” (PEs) have been studied using validated self-report measures for decades (19, 22–24). These symptoms are often non-distressing, non-impairing subthreshold forms of psychosis-like phenomena that are fairly frequently endorsed within general population samples (25, 26). In their most common form, these experiences are transient and associated with only a slightly increased risk for the later development of psychotic illness (1.5-fold increase with one occurrence of PEs) (27). However, PEs accompanied by high levels of negative affect and anxiety (28) that are distressing in nature and/or persistent over time can be associated with a substantial increase in risk for clinical psychosis [at least 10-fold (27)]. Also, individuals with an elevated risk for psychosis are also at greater risk for other forms of psychopathology and poor overall functioning (29, 30). Thus, youth with a combination of PEs and affective symptoms represent one definable group with an increased liability for developing a serious mental illness.

PEs and affective symptoms correlate in severity during both the prodromal (31) and acute (32) phases of psychosis illness. This now well-established, close phenomenological link between these two symptom categories has led to the recognition that affective symptoms likely represent a core feature of psychotic illness (33–35). Thus, a varying mixture of PEs and affective symptoms, which may change in content and severity over time, appears to represent a common form of subsyndromal psychopathology associated with increased risk for a range of poor outcomes (36, 37). Consistent with this model is the well-known mechanistic overlap between psychosis and depression -related cognitive biases (38, 39) and evidence that the presence of subsyndromal depressive symptoms alone increases risk for both the development of clinical depression (40, 41) and psychotic disorders (33, 42). Thus, interventions that target these two types of symptoms concurrently (as well as anxiety, which frequently co-occurs with both depressive and psychotic symptoms) may be of particular benefit to young people who have transdiagnostic risk factors for serious mental illness.

A key unanswered question is regarding the type of intervention that would be most effective for these youth. One approach would be to target these symptoms directly using evidence-based practices, such as CBT. An alternative approach would be to focus on a risk factor that may have a fundamental, underlying role in generating these symptoms and the associated distress. One such risk factor is social dysfunction (43–47). Impairments in social functioning are frequently present in individuals prior to the onset of serious mental illnesses (48, 49) and may increase risk for developing these illnesses (50). Although the cognitive basis of social impairment is likely complex, involving the contribution of a number of processes (51–54), impairments in day-to-day social functioning in serious mental illnesses have been repeatedly linked to deficits in social cognition (51, 55, 56), which are broadly defined as the processes that support the successful navigation of interactions with others (57).

One form of social cognition that is critical to forming relationships and supportive social networks is the set of cognitive processes underlying the understanding and awareness of the mental states of oneself and others (58, 59). Studies have found that greater self-awareness leads to improved social functioning (60). Also, the ability to accurately represent and perceive the mental states of others, i.e., mentalization or Theory of Mind (ToM) skills, correlates with community functioning, interpersonal skills, and social activity levels in individuals with serious mental illnesses (58, 61–64). In addition, related to self-awareness is the ability to feel empathy or compassion for one's own and others' suffering. Interventions that teach and improve skills in compassion have been shown to increase resilience, improve empathic accuracy and interpersonal functioning, and reduce symptom levels (65–70). Also, cross-sectional associations between higher levels of self-compassion and 1) lower levels of psychotic experiences and associated distress (71) and 2) lower levels of psychotic symptoms in individuals with schizophrenia (72) suggest that focusing on enhancing self-compassion in at-risk youth may be associated with symptom reductions. Similarly, a novel approach for enhancing mentalization skills in psychotic individuals is currently being tested (73), based on the extensive evidence for mentalization deficits in psychotic illness (74). Moreover, randomized controlled studies have found that mindfulness-focused interventions are beneficial to people with psychotic disorders, leading to decreases in symptoms and improvements in functioning (75–78). 

Therefore, in light of the critical role of processes related to understanding the self and other in successful social functioning and the evidence for impairments in these domains in individuals affected by (60, 79–81) or at risk for (82–84) serious mental illnesses, we developed and tested a mindfulness-focused intervention, designed to increase self and other awareness and compassion, for college students experiencing mild symptoms of psychosis (PEs) and/or depression. This 4-session group intervention included components of three established, evidence-based psychotherapies that focus on enhancing mindfulness (85, 86), mindful self-compassion (65, 87–89), and mentalization (78, 90, 91). Mindfulness practice emphasizes non-judgmental awareness of one's own thoughts, physical state, and the current environment (85, 86), whereas mindful self and other-compassion practice involves the combination of mindfulness with a practice of active compassion towards one's own and others' experience of suffering (65, 88, 89). Similarly, mentalization practice is based on a foundation of mindfulness that is combined with a focus on the development of a more flexible understanding of the mental states and intentions of others (90, 92). Increased awareness of one's own and others' mental states increases cognitive flexibility during the interpretation of social interactions, suggesting training in these skills could improve social functioning (93, 94).

In this single arm pilot study, we assessed whether this program was acceptable to college students and feasible to deliver on a college campus. In addition, we explored whether there was preliminary evidence for beneficial effects of the intervention on psychopathology, resilience, and social functioning.

## Methods

### Overview

Both the screening and intervention components of this program were conducted on a university campus. During the screening and recruitment process, the intervention was described as a “resilience training” workshop. Enrolled students were compensated $20 for participation in each component of the program including the screening, pre and post -intervention evaluation, and each intervention session attended.

We conducted three in-person mental health screenings at a local university over one or two days, in a high traffic area of the university, e.g., near the main cafeteria or the entry way of a group of dormitory buildings. Signs saying “free psychological screening” were hung nearby, and study staff were available to consent participants and answer questions. Students who chose to participate in the screening portion of the study signed a consent form and completed two self-report questionnaires [the Beck Depression Inventory, BDI I (95)] and the Peters et al. Delusions Inventory, PDI, (21). Individuals who met inclusion criteria for participation in the intervention (see below), and agreed to participate, were invited to complete a baseline assessment, which was scheduled for the following week and consisted of measures of symptoms (i.e., of depression, anxiety, and psychotic experiences), resilience-related capacities, and indices of social functioning. One week after the baseline assessment, participants began the 4-week intervention. Immediately following the last session of the intervention, participants repeated the assessment measures in addition to an assessment of satisfaction with the intervention. All procedures were approved by the Partners Healthcare Institutional Review Board (IRB), as well as the IRB of the participating university, and written informed consent was obtained from all subjects prior to participation.

### Participants

For the screened participants to be eligible for the intervention, they had to endorse mild to moderate depressive symptoms (BDI total score > 5) and/or psychotic experiences (PDI total score > 3) (28, 96, 97). These cut-off scores for the BDI and PDI were determined based on median values identified in similar cohorts (96, 97); we aimed to identify college students who had scores in approximately the upper half of the distribution of this sample. Potential participants were excluded if they were not proficient in English or were currently receiving psychological treatment or a prescribed medication (other than stimulants) for psychiatric reasons.

### Intervention

The intervention incorporated elements of established mindfulness, mindful self-compassion, and mentalization -based interventions and consisted of four weekly 1.5-hour group sessions. Each session involved the introduction of a new skill, an experiential exercise designed to increase understanding of that new skill, and review and assignment of home practice relevant to that skill. See **Table 1** for more details about the content and structure of each session.

**Table 1:** Overall structure and content of resilience training workshop.

**Session 1:**
- Introductions
- Welcome and orientation
- Discuss the purpose of the workshop—to increase psychological resilience
- Discuss importance of resilience—why it is particularly useful at this life stage
- Introduce skill for the week—mindfulness
- Explore what contributes to and detracts from the ability to be mindful
- Engage in experiential exercise to highlight new skill: mindful eating
- Summary—relate mindfulness to resilience
- Solicit any feedback from the group. Emphasize participants' role as collaborators
- Assign home practice: (a) 3-minute breathing exercise performed 3 times and (b) select one activity to practice mindfully
**Session 2:**
- Mindfulness exercise: self-compassion break
- Home practice review
- Review main points of previous session and restate goal of workshop
- Introduce skill for the week—self-compassion
- Explore what contributes to and detracts from self-compassion
- Engage in experiential exercise to highlight new skill: self-compassion writing exercise
- Summary—relate self-compassion to resilience
- Solicit any feedback from the group
- Assign home practice: (a) 3-minute breathing exercise performed 3 times and (b) try the self-compassion mindfulness exercise at least once
**Session 3:**
- Mindfulness exercise: mindfulness of relationships
- Home practice review
- Review main points of previous session and restate goal of workshop
- Introduce skill for the week—mentalization
- Explore what gets in the way of accurate mentalizing
- Engage in experiential exercise to highlight new skill: alternative beliefs
- Summary—relate mentalization to resilience
- Solicit any feedback from the group.
- Assign home practice: (a) 3-minute breathing exercise performed 3 times and (b) try the alternative beliefs exercise
**Session 4:**
- Mindfulness exercise: inner strength
- Home practice review
- Engage in experiential exercise to practice mentalization skills: alternative beliefs
- Consolidate and review: resilience, mindfulness, self-compassion, mentalization
- Engage in experiential exercise: self-compassion letter
- Solicit any feedback from the group. Discuss how they might use the workshop skills in the future. Discuss anything that has been meaningful for them

Sessions included 6–10 participants and were co-facilitated by 2 psychologists or 1 psychologist and a psychology intern. The students were asked to serve as active collaborators (i.e., to provide weekly feedback about what they liked and benefited from and what was less effective) and were given a “certificate of completion” following the program, which documented this collaborative role. All sessions were audiotaped to permit an independent rating of adherence to the program. Fifty percent of the sessions were evaluated using an adapted adherence scale (98) by two psychologists who were familiar with the intervention but were not group leaders. Twenty-five percent of the sessions were double coded to establish an inter-rater reliability. The one-way random intra-class correlation was 0.74 (*p* < 0.001). Ratings indicated that there was a high degree of adherence by the instructors to the protocol (mean ratings indicated 87%).

### Outcomes and Their Measures

#### Symptoms

##### Depressive Symptoms

The Beck Depression Inventory I [BDI, (95)] is the most widely used and well-validated inventory for assessing the affective, cognitive, motivational, and somatic symptoms of depression. It is a 21-item self-report scale with higher scores indicating more depressive symptoms. In this study, the internal consistency of the BDI was excellent, with α = 0.90 and 0.88 in the screened sample and in the intervention participants, respectively.

##### Psychotic Experiences (PEs)

The Peters Delusions Inventory [PDI, (21, 99)] is a 21-item self-report inventory that incorporates a multidimensional measurement of delusional beliefs and unusual experiences, with subscales measuring the distress, preoccupation, and conviction associated with those beliefs/experiences. The current study focused on 1) the number of such beliefs or psychotic experiences (PEs) endorsed and 2) the level of associated distress reported. Internal consistency was high, with α = 0.74 for the PDI Total score and α = 0.84 for the PDI Distress score in the screened sample (in the intervention sample, the sample size was too low to obtain internal consistency values).

##### Anxiety Symptoms

The Spielberger State-Trait Anxiety Inventory [STAI; (100)] is a widely used measure of trait and state anxiety; the trait scale assesses how participants typically feel in terms of anxiety-related thoughts and experiences. Responses are scored on a 4-point Likert scale ranging from 1 (“almost never”) to 4 (“almost always”) with summed scores ranging from 20 to 80. The STAI-T (the outcome examined here) displays good convergent and discriminant validity, internal consistency, and retest reliability (100). In the current study, internal consistency of the STAI-T was high, with α = 0.94 and 0.92 in the screened sample and in the intervention participants, respectively.

#### Resilience-Promoting Capacities

##### Self-Compassion

The Self Compassion Scale [SCS; (101)] is a 26-item self-report scale that measures several aspects of self-compassion: self-kindness, self-judgement, common humanity, social isolation, mindfulness, and over-identification. In the current study, the total SCS score was the outcome measure of interest, with higher scores indicating higher levels of self-compassion. This scale has demonstrated good construct validity and test-retest reliability (101) and measures aspects of adaptive psychological functioning (102). In the current study, internal consistency was high, with α = .93 and .91 in the screened sample and in the intervention participants, respectively.

##### Mindfulness

The Five Facet Mindfulness Questionnaire [FFMQ; (103)] was used to measure mindfulness. This 39-item self-report scale has six subscales; however, in the current study, we used the total score of the FFMQ as the outcome measure of interest, with higher scores indicating higher levels of mindfulness. This scale has demonstrated good reliability and construct validity (103, 104). In the current study, internal consistency was good, with α = 0.86 and 0.88 in the screened sample and in the intervention participants, respectively.

##### Mentalization

To assess one aspect or consequence of successful mentalization, we measured self-reported levels of empathy using the Interpersonal Reactivity Index [IRI; (105)]. We focused on two subscales of the IRI, which measure affective and cognitive aspects of empathy, respectively. The Empathic Concern (EC) subscale of the IRI assesses feelings of warmth, compassion, and concern for other's distress, whereas the Perspective Taking (PT) subscale assesses the ability to see things from another person's point of view. Higher scores indicate higher levels of affective or cognitive empathy. These scales have demonstrated good test-retest reliability and internal consistency (105). Internal consistency was high for the EC subscale (α = 0.81 and α = 0.80) and PT subscale (α = 0.71 and α = 0.73) for the screened sample and intervention participants, respectively.

##### Self-Efficacy

The General Self-Efficacy Scale [SES; (28, 96, 97)] is a measure designed to assess the belief in one's competence to cope with a broad range of stressful or challenging situations. The scale includes 10 items, yielding a total score between 10 and 40. Higher scores indicate higher levels of general self-efficacy. Internal consistency was high, with α = 0.92 and 0.88 in the screened sample and in the intervention participants, respectively.

#### Aspects of Social Functioning (Social Motivation, Activity, and Behavior)

##### Social Motivation

A brief self-report questionnaire was used to measure the desire to spend and the actual time spent in the company of other people [called the Time Alone Questionnaire, TAQ (106)]. For this measure, participants are asked to estimate the number of hours per day they are awake, are actively interacting with others, and would want to spend with others vs. alone. Here we focused on the percentage of waking hours that subjects would prefer to spend with others as a metric of social motivation or interest, with higher scores indicating a higher level of interest (106).

##### Social Activity

The Social Network Index [SNI, (107)] is a self-report questionnaire measuring the social activity of a subject in 12 different types of relationships. Subscales of the SNI include 1) Network Diversity (ND), the number of distinct social roles a subject reports engaging in at least once every two weeks, 2) Number of Contacts (NC), the number of people a subject regularly interacts with, and 3) Embedded Networks (EN), the number of networks in which a subject is highly active.

##### Social Behavior

We measured one form of automatic, non-verbal social behavior, social spacing or “personal space”, which is defined as the physical distance a person prefers to maintain from another person (108). Personal space-related behaviors are known to reflect certain social traits and preferences and the ability to perceive social cues (108). Both the size and the flexibility (“permeability”) of personal space for each participant was measured using the classic Stop Distance procedure (109). In this procedure, a subject stands 3 meters away from an unknown experimenter, who then slowly approaches the subject. The subject indicates when he or she becomes slightly uncomfortable (Distance 1, D1), which corresponds to the outer edge of his or her personal space “bubble.” The experimenter then continued to approach the subject until he or she became highly uncomfortable (Distance 2, D2), indicating that the subject's personal space has been entered. The ratio between D1 and D2 represents the “permeability” of the subject's personal space, with higher numbers indicating more permeable personal space. This procedure is repeated twice, with a male and a female experimenter; these two values are averaged to produce an average D1 and permeability value. This task was administered in a nonrandom subset of the intervention participants (n = 34, the subjects enrolled in the second half of the study) following optimization of the procedure in an initial group of participants.

#### Satisfaction Ratings

Following the intervention, participants completed a feedback form. In this form, participants rated on a 5-point Likert scale, from (1) “not at all” to (5) “very much” their perception of: 1) how beneficial the intervention was, 2) how useful the concepts covered were, 3) how novel the concepts covered were, and 4) if they would recommend the intervention to friends. They also ranked the intervention skills according to their relative importance to them. These ratings were collected from a subset of the intervention participants (n = 36), the second half of the sample enrolled.

### Data Analysis

To assess the feasibility of the program, we calculated the percentage of eligible students who participated in and completed the program and the average number of sessions attended. To assess acceptability, we calculated mean satisfaction ratings. We also conducted paired samples t-tests to explore where there were any pre-to-post changes in symptoms, resilience-related capacities, or social functioning. Finally, we calculated change scores from baseline to post-intervention and then conducted correlational analyses (Pearson's) for the measures that showed significant change (as indicated by the results of the paired samples t-tests), to explore whether changes in symptoms or social functioning were associated with increases in resilience-related capacities.

## Results

### Participants

The sample who volunteered to be screened consisted of 416 young adults (see **Table 2** for demographic details). These participants were on average 19.34 years old (SD = 1.3; range 18 to 23) and 65% female. Of those screened, 72 participants were enrolled in the intervention. These participants were on average 19.33 years old (SD = 1.2; range 18 to 23) and 60% female.

**Table 2:** Demographic characteristics of participants.

	Screened Sample	Intervention Sample
	N = 416	N = 72
Mean Age	19.34	19.33
Female	65%	60%
Heterosexual	77%	69%
Race		
Caucasian	52%	58%
Asian	36%	24%
African American	7%	3%
Multi-Racial	4%	3%
Other	1%	2%
US Born	73%	28%
Family Income		
>200K	18%	19%
80-200K	28%	31%
50-80K	18%	18%
25-50K	13%	14%
<25K	4%	3%

Of the 416 screened, 345 were eligible for the workshop based on their scores on the BDI and PDI (58 of these 345 students were then excluded because they were currently receiving psychiatric treatment). Of the 345 students who were eligible based on their BDI and PDI scores, 65 students (15.6%) were eligible for the workshop due to having a BDI total score of > 5 only, 80 students (19.2%) were eligible due to having a PDI total score of > 3 only, and 200 of the students (48.1%) were eligible due to having elevated scores on both measures (BDI score > 5 and PDI score > 3).

Of the 72 students enrolled in the workshop, 14 students (19.4%) had a BDI score of > 5 only, 20 students (27.8%) had a PDI score of > 3 only, and 38 students (52.8%) had elevated scores on both measures.

The 72 participants who enrolled in the intervention did not significantly differ from the screened students who did not take part in the intervention (n = 344) with respect to demographic characteristics, including age (*t =* 0.02, *p =* 0.98), gender (χ^2^ = 1.12, *p =* 0.29), sexual orientation (χ^2^ = 3.32, *p =* 0.07), race (χ^2^ = 2.64, *p* = 0.62), and location of birth (inside vs. outside the USA) (χ^2^ = 1.07, *p =* 0.30). These two groups also did not differ with respect to levels of baseline symptoms and resilience-promoting capacities (all *p*s *>* 0.05), with the exception of baseline psychotic experiences and empathic concern. Compared to students who did not participate, those who participated in the workshop had significantly higher baseline levels of psychotic experiences (*t =* 1.98, *p =* 0.048) and empathetic concern (*t* = 2.37, *p =* 0.006).

### Feasibility

**Figure 1:**
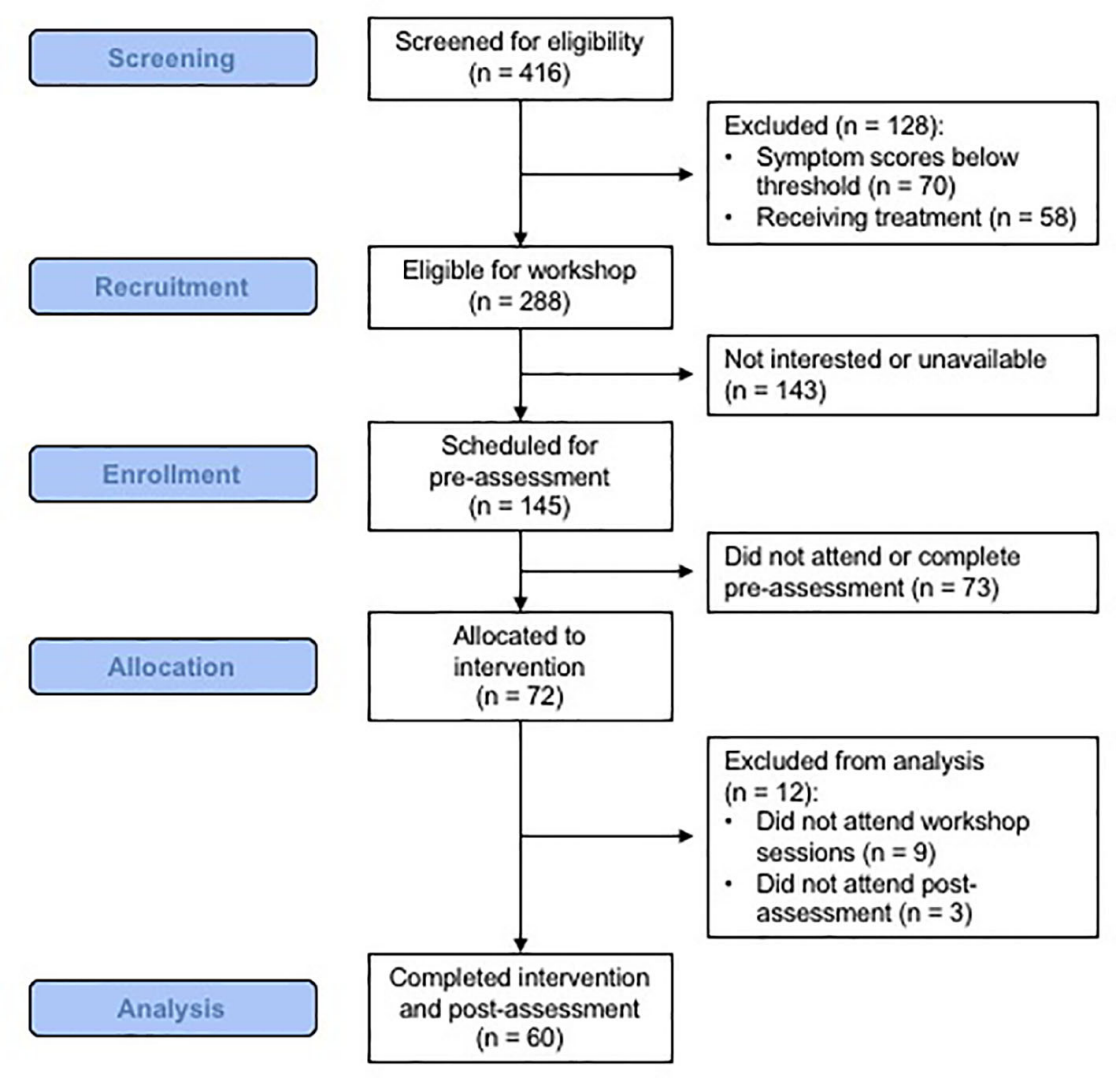
A flowchart of participant recruitment and enrollment.

Participant enrollment and flow rates indicate that it was feasible to identify, enroll, and retain college students in this program. As shown in **Figure 1**, of the 416 students screened, 69% (n = 288) had PDI and/or BDI scores that met the eligibility cut-offs of the study, and 17% (n = 72) were ultimately enrolled in the intervention. Within the screened sample, the most frequent reasons for not participating was: 1) being eligible but not being available at the time of the workshop and/or not being interested in participation (n = 143), 2) being ineligible because of having symptoms that were below the PDI and BDI thresholds (n = 70) or 3) being ineligible because of receiving treatment (n = 58). Those interested in participating were scheduled for an in-person baseline visit; 50% of those invited to that visit completed it. Participants who completed the baseline visit were then eligible to participate in the intervention. Of the 72 enrolled, 9 failed to attend any intervention sessions (13%) and 3 failed to complete the post-intervention assessment (4%). Thus, the intervention included a total of 63 participants, with 60 included in the pre-to-post assessment comparisons (see below). The participants attended on average 3.49 (SD = .76) of the 4 sessions (87% attendance). Of those who participated, 62% completed all 4 sessions, 29% completed 3 sessions, 6% attended two sessions, and 3% attended one session (91% completed 3–4 sessions).

### Acceptability

A subset of intervention participants (50% of total, n = 36, the second half of the sample) reported on their perception of the acceptability and their overall satisfaction with the program. These students indicated that the group was beneficial (M = 3.89, SD = 0.98, range: 1–5) and that the concepts were useful (M = 4.22, SD = 0.96, range: 2–5) and moderately novel (M = 3.08, SD = 0.77, range: 2–5). The majority of participants said they would recommend this intervention to a friend (M = 4.08, SD = 0.91, range: 2–5). Almost half of participants ranked Self-Compassion as the most important topic (42%), followed by Mindfulness (25%), Resilience (22%), and Mentalization (11%).

### Outcomes

Correlations, means, and standard deviations of the primary outcome variables at baseline for the 72 workshop participants are presented in **Tables 3** and **4**. At baseline, symptoms of depression, psychotic experiences, and anxiety were positively correlated with one another and negatively correlated with the resilience-related capacities, as expected.

**Table 3:** Correlation (Pearson’s r) of main study variables pre-intervention (N = 72).

Variables		1	2	3	4	5	6	7	8	9	10	11	12	13
1	Depressive Symptoms	–	.42***	.60***	.74***	-.59***	-.52***	.13	-.13	-.40**	-.09	-.06	.14	.05
2	Psychotic Experiences (PE)		–	.90***	.39**	-.28*	-.24*	.33**	-.10	-.19	-.13	-.22	-.05	.09
3	PE-related Distress			–	.56***	-.41***	-.35**	.30*	-.20	-.25*	-.18	-.26*	.02	.08
4	Anxiety Symptoms				–	-.67***	-.62***	.13	-.16	-.53***	-.20	-.16	.19	.01
5	Self-Compassion					–	.71***	-.23	.27*	.48***	.17	.15	-.15	-.03
6	Mindfulness						–	-.11	.33**	.55***	.19	.13	-.04	.02
7	Empathetic Concern							–	-.08	-.20	-.32**	-.03	-.02	.12
8	Perspective Taking								–	.25*	.20	.09	-.08	-.20
9	Self-Efficacy									–	.19	.27*	-.23	.21
10	Social Motivation										–	.04	-.16	.03
11	Social Activity											–	-.12	.46**
12	Personal Space Size												–	-.44**
13	Personal Space Permeability													–
Mean		8.36	4.54	11.40	43.17	2.95	122.76	16.21	19.41	30.35	0.31	1.89	98.67	55.89
SD		7.15	2.93	10.15	11.20	0.61	17.20	5.86	4.38	4.64	0.27	1.30	36.18	12.27

*p < 0.05, **p < 0.01, ***p < 0.001.

**Table 4:** Baseline and post-intervention values of outcome variables (N = 60).

Variable	Mean (SD)		Median (IQR)		*t*-value	95% confidence interval		*df*	*p* value	Cohen's *d*
	Baseline	Post-intervention	Baseline	Post-intervention		Lower	Upper			
**Symptoms**										
Depressive Symptoms^a^	8.10 (5.64)	6.27 (5.63)	7.00 (10.00)	4.50 (7.00)	3.05	0.632	3.035	59	**0.003**	0.39
Anxiety Symptoms	42.60 (10.09)	39.30 (9.76)	42.00 (18.00)	38.50 (14.00)	3.18	1.225	5.375	59	**0.002**	0.41
Psychotic Experiences (PE)^a^	4.57 (2.82)	3.58 (2.78)	4.00 (4.00)	3.00 (4.00)	4.13	0.507	1.460	59	**0.000**	0.53
PE-related Distress^b^	11.17 (8.72)	8.68 (8.77)	9.00 (10.00)	6.50 (11.00)	3.46	1.046	3.921	59	**0.001**	0.45
**Resilience**										
Self-Compassion	2.98 (0.59)	3.19 (0.65)	2.96 (0.81)	3.13 (1.05)	-2.93	-0.350	-0.066	58	**0.005**	0.38
Mindfulness	124.54 (17.04)	127.69 (22.17)	121.00 (25.00)	121.00 (30.00)	-1.37	-7.763	1.458	58	0.176	0.18
Empathetic Concern	16.19 (5.71)	15.64 (6.75)	17.00 (7.00)	16.50 (12.00)	1.13	-0.419	1.503	58	0.263	0.15
Perspective Taking	19.47 (4.48)	19.39 (5.01)	20.00 (6.00)	19.00 (8.00)	0.15	-1.030	1.199	58	0.880	0.02
Self-Efficacy	30.37 (4.85)	32.12 (4.79)	30.00 (9.00)	32.00 (8.00)	-3.23	-2.827	-0.664	58	**0.002**	0.42
**Social Functioning**										
Social Motivation	0.33 (0.29)	0.49 (0.22)	0.21 (0.41)	0.50 (0.28)	-4.73	-0.245	-0.099	58	**0.000**	0.62
Social Activity	1.93 (1.30)	2.29 (1.26)	2.00 (2.00)	2.00 (2.00)	2.13	-0.690	-0.021	58	**0.037**	0.28
Personal Space Size	100.48 (38.11)	87.77 (29.66)	89.00 (65.00)	85.25 (48.00)	-2.54	9.970	12.810	58	**0.016**	0.37
Personal Space Permeability	57.05 (13.08)	56.01 (12.59)	55.16 (18.24)	52.77 (18.45)	0.67	-0.314	4.922	58	0.508	0.08

a The post-intervention values were significantly skewed.

b The baseline and post-intervention values were significantly skewed. Thus, the t-test results must be interpreted with caution. SD, standard deviation; IQR, interquartile range. Significant p values (<0.05) are in bold text.

#### Changes in Symptom Levels

**Figure 2:**
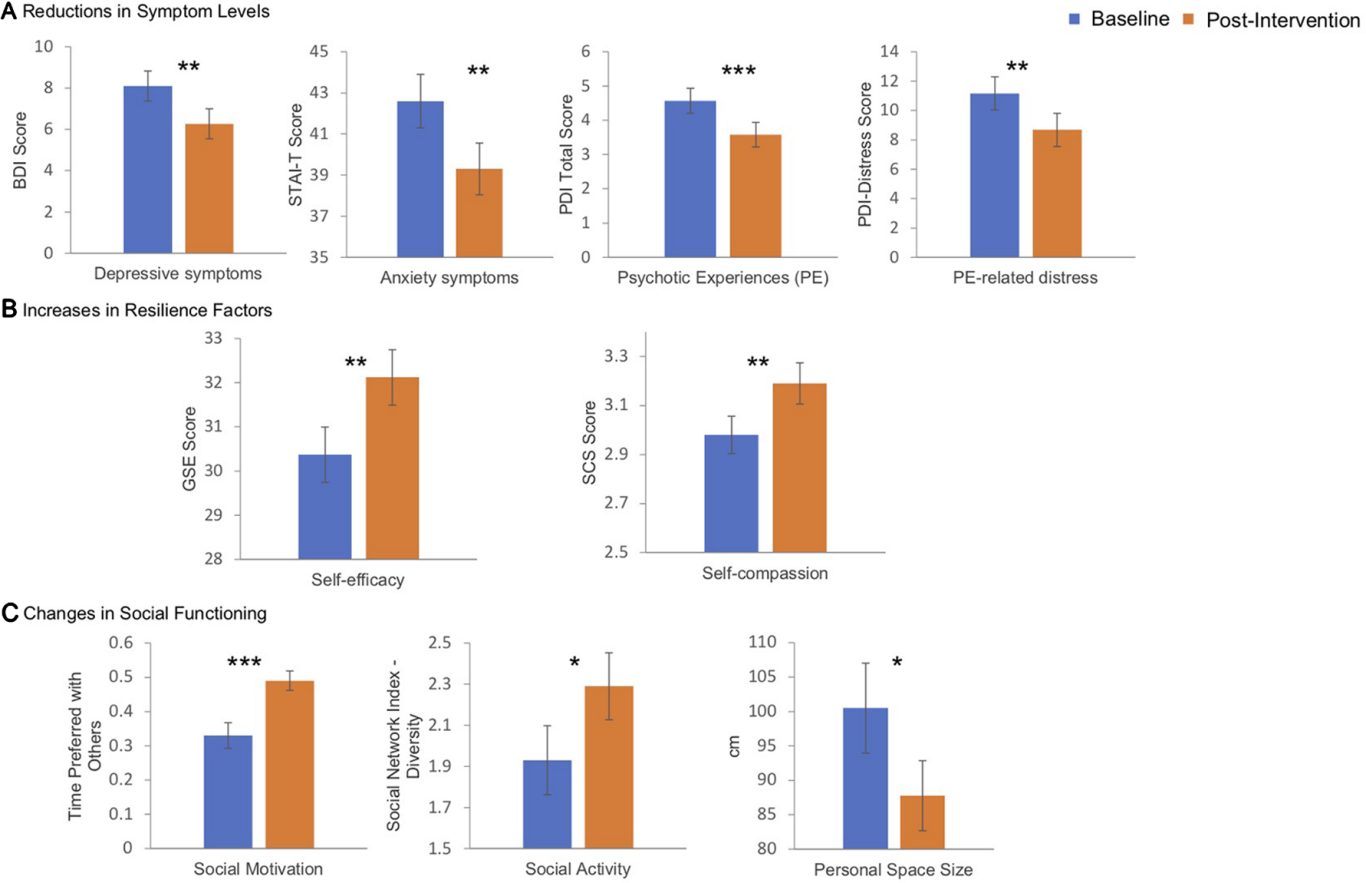
These bar plots display the reductions in symptoms of depression, anxiety and psychotic experiences, as well as in the distress associated with psychotic experiences, that followed the 4-session intervention **(A)**; these symptoms were measured using the Beck Depression Inventory, Speilberger State and Trait Anxiety Inventory (trait subscale), and the Peters et al. Delusions Inventory (Total score and Distress subscale), respectively. The intervention was also followed by significant increases in measures of resilience-related capacities, such as self-efficacy and self-compassion (measured using the Self Efficacy Scale and the Self-Compassion Scale, respectively) **(B)** and significant improvements in aspects of social functioning, including social motivation (measured using the Time Alone Questionnaire), social activity (measured with the embedded networks subscale of the Social Network Index), and personal space (measured using the Stop Distance Procedure) **(C)**. * p < 0.05; ** p < 0.01; *** p < 0.001.

Intervention participants had significantly lower levels of depression and anxiety and psychotic experiences, as well as distress associated with psychotic experiences, following the intervention compared to baseline (all p < 0.004; **Table 4**; **Figure 2A**).

#### Changes in Resilience-Related Capacities

Intervention participants exhibited significantly higher levels of self-compassion and self-efficacy following the intervention compared to baseline (all p < 0.006; **Table 4**; **Figure 2B**). There were no significant changes in the measures of mindfulness (FFMQ total score) or mentalization (EC and PT subscales of the IRI).

#### Changes in Measures of Social Functioning

Intervention participants had on average a significantly higher level of social motivation and number of embedded social networks and a significantly smaller personal space size following the intervention compared to baseline (all p < 0.04; **Table 4**; **Figure 2C**). There was no significant change in personal space permeability.

#### Associations Between Changes

**Figure 3:**
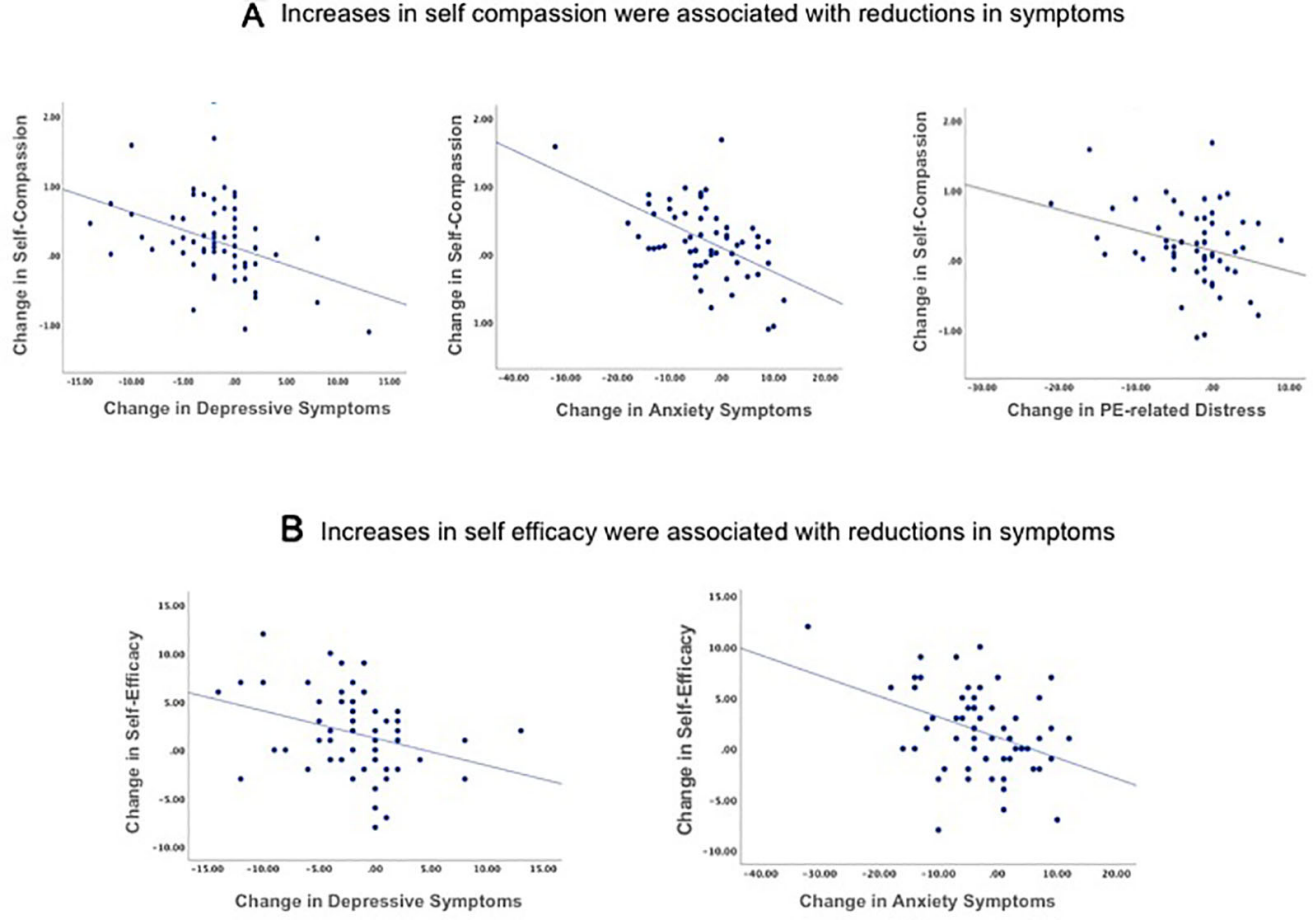
Correlations between the pre-to-post intervention improvements in self-compassion **(A)** and self-efficacy **(B)** and the pre-to-post intervention reductions in symptoms of depression, anxiety, and distress associated with psychotic experiences (PE) are displayed in these scatter plots. Increases in self-compassion and self-efficacy were correlated with reductions in symptoms (all p < 0.05). Depressive symptoms were measured using the Beck Depression Inventory, anxiety symptoms were measured using the Speilberger State and Trait Anxiety Inventory (trait subscale), and distress associated with psychotic experiences was measured using the distress subscale of the Peters et al. Delusions Inventory.

We assessed whether the observed reductions in symptoms or improvements in social functioning were associated with any of the increases in resilience-related capacities. To do this, we calculated change scores (change from baseline to post-intervention) only for the scales that showed significant change. The increases in self-compassion from baseline to post-intervention were significantly correlated with the reductions in depressive (*r* = -0.43, *p =* 0.001) and anxiety (*r* = -0.53, *p <* 0.001) symptoms and with the reductions in distress associated with psychotic experiences (*r* = -0.30, *p =* 0.02), but were not correlated with the reduction in the number of psychotic experiences (*r* = -0.15, *p =* 0.21) (**Figure 3A**). The increase in self-efficacy from baseline to post-intervention was correlated with the reductions in depressive (*r* = -0.32, *p = .*03) and anxiety (*r* = -0.39, *p =* 0.003) symptoms (**Figure 3B**), but not with the changes in psychotic experiences (*r* = -0.15, *p =* 0.24) or the distress associated with those experiences (*r* = -0.21, *p =* 0.10). The changes in social functioning following the intervention were not correlated with any of the pre-post changes in resilience-related capacities (self-compassion and self-efficacy).

#### Effects of Adherence

Lastly, participants who attended all four of the sessions (with 100% adherence) experienced a greater reduction in depressive symptoms following the intervention than those with a lower level of attendance (t = -2.06, p = 0.04). There were no other differences in the degree of change in the measures of interest between the participants who showed 100% adherence (n = 39) compared to those who did not (n = 21).

## Discussion

### Summary of Findings

This single arm pilot study demonstrates that a college campus-based screening for subclinical psychopathology, and 4-week intervention focused on exposing at-risk college students to the concepts and skills of mindfulness, self-compassion, and mentalization, is feasible and acceptable to participants, since 91% of participants attended at least 3 of the 4 sessions, and the majority rated it as beneficial and would recommend it to a friend. In addition, preliminary evidence for benefits of the intervention were observed; reductions in depression, anxiety, and psychotic experiences and distress associated with psychotic experiences, as well as increases in resilience-related capacities (self-compassion and self-efficacy) and positive changes in several aspects of social functioning (social motivation, social network size, and comfort with the physical proximity of others), followed the intervention. Moreover, the magnitude of increases in resilience-related capacities correlated with the magnitude of decreases in symptoms of depression, anxiety, and distress associated with psychotic experiences, providing a basis for further assessment of these potential relationships in a follow-up randomized controlled trial.

### Feasibility and Acceptability

The high retention numbers and the students' favorable assessments of the program suggest that many college students are interested in improving their emotional health and are willing to spend time on this goal. The fact that 70% of those screened were eligible for the program likely resulted from a combined effect of our liberal inclusion criteria [designed to capture the upper 50% of the typical distribution of BDI and PDI scores in this population (96, 97)] and self-selection for the screening procedure, which was voluntary. This “wide net” approach to screening likely contributed to the robust enrollment and retention despite the inevitable attrition (when trying to engage busy college students in a non-required activity) that occurred prior to the start of the intervention. We also speculate that the explicit framing of the intervention as a resilience workshop (rather than as a treatment), in which feedback was solicited from the participants at every session, may have had a de-stigmatizing effect, potentially also facilitating enrollment and retention rates. However, the fact that the students were compensated for their participation (albeit a modest amount for these predominantly middle-to-upper-middle class students) may have played a role in these participation rates. The feasibility of this or similar programs when delivered without explicit incentives or using non-financial ones (e.g., class credit) can be examined in future studies.

### Changes in Symptoms and Resilience-Promoting Capacities Following the Intervention

There were significant reductions in depressive and anxiety symptoms and in the number of psychotic experiences, and in the distress associated with these experiences, following the intervention. The reductions in affective symptoms following the intervention were correlated with increases in self-compassion and self-efficacy. These results are consistent with previously reported cross-sectional associations between greater levels of self-compassion and lower levels of depression and anxiety (110). Similarly, a randomized controlled study of the effects of Mindful Self-Compassion (MSC), an 8-session group intervention that focuses specifically on enhancing self-compassion (from which the self-compassion exercises of our program were adapted), showed that treatment with MSC is associated with reductions in symptoms of depression and anxiety in a non-clinical sample (66). It has been hypothesized that self-compassion reduces negative affect by increasing a person's ability to tolerate stress and maintain equanimity during difficult experiences, including interpersonal conflicts and perceived failures, perhaps by improving the capacity to regulate emotions and experience positive affect (70, 111). This effect of self-compassion may be particularly helpful for college students. Prior studies have shown that college students with higher levels of self-compassion are better able to manage academic set-backs and social stress, are more willing to try again following academic failures, and experience less homesickness and depression than those with lower levels of self-compassion (70, 88, 112). The high acceptability ratings for the self-compassion portion (rated as the most important concept learned) of the workshop suggests that the self-compassion module of the intervention may have been the most effective of the three main components of the program. Due to its intrinsic appeal, self-compassion may have been the most deeply acquired skill of those introduced during the 4-session time frame.

In contrast to self-compassion, we did not observe any changes in our measures of mindfulness and mentalization following the intervention. Although very brief mindfulness-based interventions have been shown to have positive effects in adolescents (113, 114), our intervention may not have been long enough to produce measurable changes (as reflected by changes in scores on the selected self-report measure) in these skills, which may require a period of training longer than four weeks, particularly in college students who typically have numerous competing demands. Unlike in conventional mindfulness interventions (e.g., mindfulness based cognitive therapy, which is comprised of 8 two-hour sessions with daily practice), participants in this program were not required to engage in extensive exercises between sessions (the mindfulness exercises were recommended only) because we felt that such a requirement would reduce retention levels and overall feasibility of the intervention; our initial piloting suggested that the students were unlikely to complete such assigned “homework”.

Another possible reason for the absence of changes in mindfulness and mentalization following the workshop is that any such changes may not have been associated with change in our measure of mindfulness and in our indirect measure of mentalization, the Interpersonal Reactivity Index (IRI), and thus may have gone undetected. The IRI, a self-report measure of empathy, which asks the participant to assess how he or she generally tends to react in certain situations, may not be sensitive to subtle, short-term changes in social perception and automatic attitudes about others' intentions.

Lastly, we also observed changes in social motivation (an increase in the percentage of time that a participant preferred to spend with others), social network size and complexity (an increase in the number of “embedded” networks), degree of comfort with the physical proximity of others (a decrease in the size of personal space) following the intervention. None of these changes correlated with the changes in resilience-related capacities. However, it is possible that some components of the intervention, or a combination of them, are associated with increases in psychological comfort with the self and others and in the ability or desire to spend time with others, perhaps in part due to an increased capacity to manage difficult or conflicting emotions or experience positive affect during interpersonal interactions. Follow-up work (i.e., a randomized controlled trial) can systematically test this hypothesis.

### Limitations

Our findings should be considered in light of several limitations of this pilot study. First, given the study's single-arm design, it remains unknown whether the changes in symptoms and resilience-related capacities we observed following the intervention were causally related to the specific skills taught or were instead due to non-specific, group support-related effects (or to the passage of time). A follow-up randomized controlled trial will test these alternative possibilities. Related to this issue, the question of whether the program is associated with any reductions in long-term levels of risk for serious mental illness remains unaddressed by this study and must be examined in future, longitudinal analyses.

Second, both a strength and limitation of the study is that the intervention was brief, lasting only four sessions. This design was chosen to increase the feasibility and acceptability of the intervention for college students, who tend to have limited free time and typically change their schedules on a semester-by-semester basis. The majority of participants of the program did not suggest lengthening the intervention.

Third, although this study employed well-validated and commonly-used scales, it largely relied upon self-report measures. Although this may have introduced subjective biases, the benefits of low-cost, easily administered, reliable, and valid measures at this stage of this line of research outweighed, in our view, the potential gains of additional information and diagnostic precision provided by interview-based research instruments. Given that a central goal of this work is to develop an approach that can be implemented in the “real world” at a wide range of institutions of higher education and community settings, we chose to prioritize feasibility over comprehensiveness.

Fourth, the aim of this program is to identify college students who have some risk for serious mental illness. However, because of the lack of available information about the long-term outcomes of individuals with mildly-to-moderately elevated scores on the two measures used in the current study to identify at-risk individuals, we cannot estimate the precise level of risk for serious mental illness our participants had. However, related data suggest that individuals with persistent or recurring psychotic experiences have at least a 10-fold increase in risk for developing clinical psychosis, which increases in proportion to the degree of persistence of these symptoms in a dose-dependent fashion (27). This level of risk is in the range of that of first-degree relatives of people with psychotic disorders (115). However, the exact risk level(s) for developing psychosis or another serious mental illnesses that is associated with varying levels of subclinical psychotic or depressive symptoms, or both symptoms in combination, is unknown and requires further study.

### Future Directions

This pilot study of an approach that engages a specific subset of college students with a small-to-moderate risk for developing a serious mental illness can provide a basis for additional studies of a variety of potentially protective interventions for vulnerable individuals in this age group, ranging in intensity from monitoring only (to facilitate early detection if clinical symptoms arise) to a brief exposure to resilience-building skills (e.g., the intervention described here) to more intensive, individually-tailored treatments targeting symptoms that are beginning to affect daily functioning. Ongoing longitudinal follow-up assessments of these at-risk youth may identify the characteristics that are most closely linked to poor outcomes in this population, permitting a more precise assessment of risk and selection of the appropriate intervention. Predictors of objective functional outcomes, including academic performance, usage of mental health services, and school retention, may be particularly useful and appropriate for assessing transdiagnostic risk levels in this population.

## Conclusions

In summary, we have described the rationale, methods, and evidence for feasibility and acceptability of a college campus-based early detection and prevention program aimed at reducing risk for the later development of disabling psychopathology. The program delivers a brief intervention focused on enhancing emotional resilience in vulnerable youth. The results of this pilot study suggest that this program warrants further study, as it may represent a novel, relatively non-stigmatizing approach for providing protective, resilience-boosting skills to young adults.

## Data Availability Statement

The final datasets generated from this study are available on request to the corresponding author.

## Ethics Statement

The studies involving human participants were reviewed and approved by Partners Healthcare, Boston Massachusetts. The participants provided their written informed consent to participate in this study.

## Author Contributions

The study was designed by DH, CC, CL, BS, MN, and AB. The intervention was administered by CC, AB, MN, and AP-B. The study procedures were overseen by LL, LN, CL, WD, AB, and DH. The data were analyzed by BS and WD. The manuscript was written by DH, BS, AP-B, AB, WD and CC. All authors have reviewed the manuscript and approved the final version.

## Funding

This work was supported by the Sidney R. Baer, Jr Foundation.

## Disclaimer

This work was prepared while BS was employed at the Massachusetts General Hospital/Harvard Medical School; the opinions expressed in this article are the author's own and do not reflect the view of his current employer, the National Institutes of Health, the Department of Health and Human Services, or the United States government.

## Conflict of Interest

The authors declare that the research was conducted in the absence of any commercial or financial relationships that could be construed as a potential conflict of interest.
